# Connexin 30 (*GJB6*) deletion as a cause of a false positive sweat test result

**DOI:** 10.1007/s00431-025-06220-7

**Published:** 2025-06-12

**Authors:** Anna Rossell, Aleix Soler-Garcia, Loreto Martorell, Maria Antònia Claveria, Laura Valero, Sílvia Rodríguez, Cèlia Badenas, Maria Cols-Roig

**Affiliations:** 1 Pediatrics Department, Hospital Sant Joan de Déu, Passeig de Sant Joan de Déu 2, 08950 Esplugues de LlobregatBarcelona, Spain; 2 Infectious Diseases and Microbiome Research Group, Institut de Recerca Sant Joan de Déu, Barcelona, Spain; 3 Departament de Cirurgia i Especialitats Medicoquirúrgiques, Facultat de Medicina i Ciències de La Salut, Universitat de Barcelona, Barcelona, Spain; 4 Department of Genetic and Molecular Medicine, Hospital Sant Joan de Déu, Barcelona, Spain; 5 Department of Pediatric Otorhinolaryngology, Hospital Sant Joan de Déu, Barcelona, Spain; 6 Pediatric Pulmonology Department and Cystic Fibrosis Unit, Hospital Sant Joan de Déu, Barcelona, Spain; 7 Biochemistry and Molecular Genetics Department, Hospital Clínic de Barcelona, IDIBAPS, University of Barcelona, Barcelona, Spain; 8 Centro de Investigación Biomédica en Red en Enfermedades Raras, Instituto de Salud Carlos III, Barcelona, Spain

**Keywords:** Sweat test, Connexin 30, *GJB6*, And Neurosensory deafness

## Abstract

The sweat test (ST) is the gold standard for the diagnosis of cystic fibrosis. There are several reports in the literature regarding conditions that are known to be associated with a false positive result. The aim of this article is to describe a previously unreported cause of a false positive ST. An observational, cross-sectional single-center study was performed. We recruited three patients with a neurosensory deafness caused by a deletion in both alleles of connexin 30. The first-degree relatives of these three patients with hearing impairment due to other mutations were also included. A ST was performed in all the selected cases. Among the three patients with a deletion in both connexin 30 alleles, two had a positive ST, whereas the third patient had a close-to-positivity borderline result (57 mmol/L). Moreover, there were no positive sweat tests in individuals with other mutation patterns.

*Conclusion*: Patients with affection of both alleles of connexin 30 were the only ones to show a positive ST, which may translate to a higher risk of hyponatremic dehydration. The reason for the ST positivity remains unclear and may be related to the fact that connexin 30 plays a role in modulating other molecules in both the inner ear and sweat glands.

**Table:** 

**What is Known:** • *The sweat test is the gold standard for the diagnosis of cystic fibrosis. However, the causes of false positives in the test are increasingly recognized.*	
**What is New:** • *This study describes a previously unreported cause of a false positive sweat test. Three patients with homozygous mutations in the connexin 30 gene are described. All of them had an abnormal sweat test, and two of them presented with severe hyponatremic dehydration.*

**What is Known:**

• *The sweat test is the gold standard for the diagnosis of cystic fibrosis. However, the causes of false positives in the test are increasingly recognized.*

**What is New:**

• *This study describes a previously unreported cause of a false positive sweat test. Three patients with homozygous mutations in the connexin 30 gene are described. All of them had an abnormal sweat test, and two of them presented with severe hyponatremic dehydration.*

## Introduction

The sweat test (ST) consists of a measurement of chloride concentration and conductivity in a sample of sweat. The test is mainly used as the gold standard for the diagnosis of cystic fibrosis (CF). CF is the most common life-threatening autosomal recessive disease in the Caucasian population, due to a multisystem disorder caused by mutations in the gene encoding the CF transmembrane conductance regulator (CFTR), which encodes a chloride channel [1]. When a patient presents a positive newborn screen, clinical symptomatology suggesting CF, or a positive family history, a diagnosis of CF can be made if the ST is pathological. An ST with a chloride concentration greater than 60 mmol/L is considered pathological, whereas values between 30 and 60 mmol/L are borderline results [2].

However, there are several reports in the literature regarding conditions that are known to be associated with false positive or false negative ST results. The list of these conditions has grown significantly over time. Some examples include malnutrition, some metabolic disorders (such as mucopolysaccharidosis, type I fucosidosis, or glycogen storage diseases), dermatological diseases (as congenital ectodermal dysplasia or atopic dermatitis), various endocrine disorders (e.g., panhypopituitarism, Addison disease, isolated adrenocorticoid deficiency, vasopressin-resistant diabetes insipidus, hypothyroidism, and hypoparathyroidism), and carbonic anhydrase XII mutations [3, 4].

In this article, we describe a previously unreported cause of false-positive ST: we identified three patients with a mutation in both alleles of the Gap Junction Beta (*GJB*) 6 gene (encoding connexin 30, which is responsible for hearing impairment) in which no other potential causes of a positive result were found.

Globally, the prevalence of congenital hearing impairment is 1–3 in 1000 children at birth or during early childhood [5]. Nonsyndromic hearing impairment (NSHI) is the most common form of neurosensory deafness, accounting for almost 70% of inherited hearing impairments. The majority of NSHI cases have an autosomal recessive pattern of inheritance [6].

The connexin gene family is the most prevalent gene that contributes to NSHI. Connexins are membrane proteins expressed throughout the body, including skin, cochlea, or muscles. Six connexins form a connexon, which, when aligned with another, allows the formation of gap junctions that allow direct cell-to-cell communication [7].

Studies in European and Asian populations have identified pathogenic variants in *GJB2* (encoding connexin 26) and *GJB6* (encoding connexin 30) as the major contributors to autosomal recessive NSHI (ARNSHI). Specifically, GJB2-c35 delG is the most prevalent variant (20–50%) found in cases of ARNSHI. The GJB6-D13S1830 deletion was identified in up to 9.7% of cases and thus is the second-largest contributor to the genetic etiology of NSHI in Western European populations, either with homozygous presentation or when present in addition to a *GJB2* mutation on the opposite allele [6, 8–11]. GJB-6 deletions remove large segments of the gene and adjacent regulatory regions, even affecting the neighboring GJB2 gene also, due to disruption of shared cis-regulatory elements [12, 13]. The exact global prevalence of deafness caused by two pathogenic variants involving GJB6 is difficult to estimate. However, studies suggest that GJB6 deletions account for approximately 5–9% of cases of ARNSHI [6, 14].

On the other hand, mutations in the skin-expressed connexin genes *GJB2* (connexin 26), GJB3, *GJB4* (connexin 31 and 30.3), *GJB6* (connexin 30), and Gap Junction Alpha 1 (connexin 43) have been linked to human hereditary diseases affecting both the epidermis and cochlea [7].

## Material and methods

This was an observational, cross-sectional single-center study.

The first case was a 22-month-old female child with neurosensory deafness due to a mutation in homozygosity of both *GJB6* alleles. She required multiple admissions due to hyponatremic dehydration. Due to this, a ST was performed in the context of clinical stability and after stopping intravenous rehydration, which was positive. CF was ruled out since she did not present other symptoms compatible with CF, her neonatal screening was negative, and she had a genetic test with no mutations in CFTR.

Four months later, a 13-month-old female child with neurosensory deafness due to a mutation in the compound heterozygosity of both *GJB6* alleles was also admitted due to a hyponatremic dehydration. Considering the previous case, a ST was also performed once the dehydration had been solved and while not being under rehydration therapy, resulting in a chloride concentration of 57 mmol/L. Similarly, no other symptoms of CF were observed, and no mutations were detected in the CFTR gene.

Following these findings, the hospital’s genetic database was accessed in order to search for other patients with neurosensory deafness caused by a mutation in homozygosity or compound heterozygosity for connexin 30. Although no other pediatric patients were found, we identified an adult man with deafness with a mutation in the compound heterozygosity of both *GJB6* alleles (third case). This patient was examined in 2009 as part of a genetic study of his daughters, who also have deafness.

The first-degree relatives of these three patients with hearing impairment due to a mutation in both *GJB6* alleles were also included. Informed consent was obtained from each participant. This study was approved by the Hospital Sant Joan de Déu Ethics Committee (Esplugues, Spain, refPIC-115–24) and was done in accordance with the Declaration of Helsinki. Informed consent was obtained from the individual participants included in the study or their parents.

A ST was performed in all the selected cases according to current recommendations [15]. ST is a noninvasive test that is used for the determination of chloride in sweat. In these cases, iontophoresis was performed with the Webster Sweat Inducer–Wescor® system. The sweat sample was collected by Macroduct® system and immediately manipulated to determine conductivity via Sweat-Check 3120, Wescor®, and chlorimetry via Chloride Analyzer 926, Sherwood Scientific Ltd.

In cases 1 and 2, genetic tests were performed in order to rule out mutations in the CFTR gene. Initially, the Elucigene CF-EU2 kit test was done. Then, a direct sequencing of the coding region of the gene was performed. In addition, deletions/duplications of the CFTR gene were analyzed by Multiplex Ligation-dependent Probe Amplification (MLPA) using the SALSA MLPA P091D2 kit (MRC Holland®). The results were obtained in an automatic sequencer ABI3130. This technique detects more than 99% of mutations in the CFTR gene [16]. On the other hand, deletions in the *GJB6* gene were obtained by using the SALSA MLPA P163-GJB-WFS1-POU3 F4 kit (MRC Holland®) and analyzed with the Coffalyser program.

The Mann–Whitney *U* test was used to compare medians between groups of patients. The statistical analysis was performed via SPSS v.24, with statistical significance defined as a *p*-value of < 0.05.

## Results

We recruited three patients with a neurosensory deafness caused by a mutation in both alleles of connexin 30. A ST was also performed in their five relatives with deafness caused by other mutations. The results are described in Table 1. Among the three patients with a deletion in compound heterozygosis of connexin 30, two had a positive ST, whereas the third patient had a close-to-positivity borderline result (57 mmol/L). Moreover, there were no positive sweat tests in individuals with other mutation patterns. Patients with a deletion in both connexin 30 alleles presented increased chloride concentrations (*p*-value 0.036) and conductivity (*p*-value 0.035).

**Table 1:** Descriptive features of patients with deafness in which a ST was performed

Patient	Index case	Age	Sex	Mutation pattern	Sweat test		
					Chloride	Conductivity	Interpretation
**Case 1**	Yes	22 months	Female	*GJB6* homozygosis: [del(GJB6-D13S1830)]	67 mmol/L	80 mmol/L	Abnormal
**Case 2**	Yes	13 months	Female	*GJB6* compound heterozygosis: [del(**GJB6**-D13S18**30**) + del(**GJB6**-D13S18**54**)]	57 mmol/L	67 mmol/L	Borderline
**Case 3**	Yes	48 years	Male	*GJB6* compound heterozygosis: [del(GJB6-D13S1854) + del(GJB6-D13S1830)]	65 mmol/L	86 mmol/L	Abnormal
**Case 4**	Case 1 brother	5 years	Male	*GJB6*/*GJB2* in heterozygosis: [del(**GJB6**-D13S1830) + **GJB2**-c.35 delG]	10 mmol/L	44 mmol/L	Normal
**Case 5**	Case 1 father	35 years	Male	*GJB6*/*GJB2* in heterozygosis: [del(**GJB6**-D13S1830) + **GJB2**-c.35 delG]	23 mmol/L	46 mmol/L	Normal
**Case 6**	Case 1 mother	38 years	Female	*GJB6*/*GJB2* in heterozygosis: [del(**GJB6**-D13S1830) + **GJB2**-c.35 delG]	12 mmol/L	45 mmol/L	Normal
**Case 7**	Case 3 daughter	14 years	Female	*GJB6* heterozygosity: [del(GJB6-D13S1854)] + unknown mutation	20 mmol/L	43 mmol/L	Normal
**Case 8**	Case 3 daughter	14 years	Female	*GJB6* heterozygosity: [del(GJB6-D13S1854)] + unknown mutation	25 mmol/L	45 mmol/L	Normal

Patient 1 is a 22-month-old female child with severe hearing impairment due to a mutation in homozygosis of both *GJB6* alleles [del(GJB6-D13S1830)]. She is a bilateral cochlear implant recipient. Both the patient’s parents and her brother (cases 4–6) experienced hearing impairment due to a combination of a mutation in the heterozygosity of *GJB2* [(GJB2-c.35 delG)] and *GJB6* [del(GJB6-D13S1830)].

The patient visited the emergency department for vomiting, diarrhea, and fever for 48 hours. A blood test revealed metabolic acidosis; intravenous rehydration was started, and the patient was admitted. During hospitalization, a more thorough anamnesis revealed that she had been admitted four times in one year because of hyponatremia (up to 113 mmol/L) in the context of vomiting and diarrhea. Previously, additional studies revealed no impairments in sodium fractional excretion or in cortisol and renin levels. As part of the study, a ST was performed, which yielded a positive result on two occasions (with a chloride concentration of 67 mmol/L and conductivity of 80 mmol/L). A genetic test ruled out CF and carbonic anhydrase XII mutations. She never presented with respiratory symptoms or signs of pancreatic insufficiency. A ST was also performed on her parents and brother, who were negative (Table 1).

Patient 2 is a 13-month-old child with severe neurosensory deafness due to a mutation in the compound heterozygosity of *GJB6* [del(GJB6-D13S1830) and del(GJB6-D13S1854)] with no family history of deafness. Her father is a carrier in heterozygosity of the mutation del(GJB6-D13S1830) and her mother is a carrier in heterozygosity of the mutation del(GJB6-D13S1854).

The patient was admitted directly to the intensive care unit (ICU) due to hypovolemic shock caused by uncontrollable vomiting and refusal to eat for 12 hours in the context of high ambient temperature. Initially, a blood test revealed metabolic acidosis and hyponatremia of 120 mmol/L. After stabilization, and owing to a similar case (explained above) that presented with the same mutation, a ST was performed showing close-to-positivity results (chloride of 57 mmol/L and conductivity of 67 mmol/L). Moreover, a genetic test was performed and revealed no mutations related to CF. During 18 months of surveillance, she did not have any respiratory or gastrointestinal symptoms.

Patient 3 is a 48-year-old man with neurosensory deafness caused by a mutation in the compound heterozygosity of *GJB6* [del(GJB6-D13S1830) and del(GJB6-D13S1854)] who had been studied in 2009 after having had twins with hearing impairment (cases 7–8) due to a *GJB6* mutation in heterozygosity. After observing the findings explained above, the patient was recruited and a ST was performed, showing a positive result (chloride of 65 mmol/L and conductivity of 86 mmol/L). A thorough anamnesis was performed, and neither the patient nor his parents remembered any episode of dehydration. Moreover, the man — in his fifth decade of life — was otherwise healthy, presenting only with hearing impairment, and without any history of respiratory manifestations, gastrointestinal symptoms, infertility, or other clinical features suggestive of CF. Considering that CF is a progressive, severe, and multisystemic condition where the patient exhibited no related symptoms, the likelihood of CF was deemed to be very low. Thus, after observing the relationship between connexin 30 mutation and positive ST, a genetic test to rule out CF was not performed.

## Discussion

As previously mentioned, connexins are expressed not only in the inner ear, but also in other tissues such as sweat glands and the epidermis. There are multiple types of connexins (such as 26, 30, 31, and 43), and they tend to show overlapping expression patterns, and connexones formed by different types of connexins exist [17].

The ways in which connexins interact with each other and with other molecules are complex. They have paracrine actions, regulating, for instance, calcium and sodium channels [17–19]. They also have mechanical functions, such as allowing the contraction of the myoepithelial cells of the sweat glands, thus enabling the excretion of sweat [20]. This wide range of functions depends on subtle factors such as the position of the molecule on the cell surface or how they are placed in relation to other structures on which they exert their effect. Indeed, different types of mutations in connexin alleles affect how and where their molecules are expressed in the cells, as well as how they are transported within them [10]. This translates into a great variability at the phenotypic level as well. For example, loss-of-function mutations are more related to nonsyndrome deafness, whereas gain-of-function mutations are described in diseases involving both skin and deafness [21].

Although connexins are also expressed in sweat glands, the literature concerning this phenomenon is scarce. In this sense, it is important to highlight a paper describing some patients with connexin 26-related genes [22]. The authors examined histology and in vitro sweat production among members of families affected by R143W, a *GJB2* mutation. They reported that both heterozygous and homozygous participants had a significantly thicker epidermis. Interestingly, only homozygotes presented relatively high concentrations of sodium and chloride in the sweat, which may be interpreted as a biological disadvantage.

The reason why mutations in connexins could explain the relatively high concentrations of chloride and sodium in sweat is not clear. It is known that connexin 30 plays a role as a modulator of the activity of epithelial sodium channels (ENaC) via multiple pathways, such as the paracrine secretion of ATP or glutamate or the promotion of clathrin-mediated ENaC endocytosis [23]. In relation to this fact, Sipos et al. observed that mice with connexin 30 deficiencies had a reduced natriuretic capacity in response to hypertension, as their ability to regulate sodium reabsorption was limited [19].

These observations emphasize that connexins play a role in regulating sodium transport but would not justify a positivity in the ST. In this regard, the intrinsic actions of connexin 30 are particularly complex and are closely linked to the expression of connexin 26 in both inner ear and sweat glands. What is more, it has been defined that deletions of the *GJB6* gene directly affect the expression of connexin 26 [13]. More specifically, Common et al. showed a case of a deaf child with a del(GJB6-D13S1830) and GJB2-c.35 delG constellation in which a skin biopsy was performed. The authors compared his biopsy results with those of a healthy control and a patient with a heterozygous mutation in the connexin 26 gene. They observed that the case patient, but not the others, showed a marked decrease in the expression of connexin 26 in the duct, but not in the excretory portion of the sweat gland, without affecting the expression of connexin 30 itself — as well as an alteration of keratin expression, which acts supporting many other molecules [24].

In conclusion, although unable to give a definitive explanation, we found that all patients with a mutation affecting both alleles of connexin 30 presented concentrations of chloride in sweat higher than 55 mEq/L. However, those patients with genetic deafness explained by other allelic constellations presented with normal ST. It should be emphasized that these results may be relevant from a clinical point of view. Patients with these mutations could be exposed to a greater risk of dehydration during the first months of life, especially during the hot months in warm countries. In fact, two of the patients described in this report experienced episodes of hyponatremic dehydration that required repeated hospital admissions, including stays in the ICU. The third patient is an adult, and there is no evidence that he had suffered similar episodes. In this regard, the clinical impact of this mutation may vary between individuals. However, it does appear to be more significant in younger patients, probably due to a higher body surface area-to-volume ratio, a greater total body water content, and a reduced voluntary access to fluid intake. Additionally, external factors such as environmental heat could also play an important role—something especially relevant in the current context of global warming.

To the best of our knowledge, this is the first study in which a mutation in connexin 30 has been described as a possible explanation for the positivity of a ST in patients with severe deafness. The most important limitation of our study is the small sample size. In this sense, we think that a multicenter study should be performed to confirm this relationship.

## Data Availability

No datasets were generated or analysed during the current study.

## Abbreviations

ARNSHI: Autosomal recessive NSHI
CF: Cystic fibrosis
CFTR: Cystic fibrosis transmembrane conductance regulator
ENaC: Epithelial sodium channels
*GJB*: Gap junction beta
ICU: Intensive care unit
MLPA: Multiplex ligation-dependent probe amplification
NSHI: Nonsyndromic hearing impairment
ST: Sweat test

## References

[1] Farrell PM, White TB, Ren CL, et al. (2017) Diagnosis of cystic fibrosis: consensus guidelines from the Cystic Fibrosis Foundation. J Pediatr 181S:S4-S15.e1 10.1016/j.jpeds.2016.09.064 28129811

[2] Turcios NL (2020) Cystic fibrosis lung disease: an overview. Respir Care 65(2):233–251 31772069 10.4187/respcare.06697

[3] Feldshtein M, Elkrinawi S, Yerushalmi B et al (2010) Hyperchlorhidrosis caused by homozygous mutation in CA12, encoding carbonic anhydrase XII. Am J Hum Genet 87(5):713–720 21035102 10.1016/j.ajhg.2010.10.008 PMC2978943

[4] Guglani L, Stabel D, Weiner DJ (2015) False-positive and false-negative sweat tests: systematic review of the evidence. Pediatr Allergy Immunol Pulmonol 28(4):198–211 35923002 10.1089/ped.2015.0552

[5] Mehra S, Eavey RD, Keamy DG (2009) The epidemiology of hearing impairment in the United States: newborns, children, and adolescents. Otolaryngol Head Neck Surg. 140(4):461–472. https://pubmed.ncbi.nlm.nih.gov/19328331/. Accessed 10 March 2025 10.1016/j.otohns.2008.12.022 19328331

[6] Snoeckx RL, Huygen PLM, Feldmann D et al (2005) GJB2 mutations and degree of hearing loss: a multicenter study. Am J Hum Genet 77(6):945–957 16380907 10.1086/497996 PMC1285178

[7] Avshalumova L, Fabrikant J, Koriakos A (2014) Overview of skin diseases linked to connexin gene mutations. Int J Dermatol 53(2):192–205 23675785 10.1111/ijd.12062

[8] Del Castillo FJ, Rodríguez-Ballesteros M, Álvarez A et al (2005) A novel deletion involving the connexin-30 gene, del(GJB6-d13s1854), found in trans with mutations in the GJB2 gene (connexin-26) in subjects with DFNB1 non-syndromic hearing impairment. J Med Genet 42(7):588–594 15994881 10.1136/jmg.2004.028324 PMC1736094

[9] Wonkam ET, Chimusa E, Noubiap JJ, Adadey SM, Fokouo JVF, Wonkam A (2019) GJB2 and GJB6 mutations in hereditary recessive non-syndromic hearing impairment in Cameroon. Genes (Basel) 10(11) 10.3390/genes10110844 PMC6895965 31731535

[10] Marlin S, Feldmann D, Blons H et al (2005) GJB2 and GJB6 mutations: genotypic and phenotypic correlations in a large cohort of hearing-impaired patients. Arch Otolaryngol Head Neck Surg 131(6):481–487 15967879 10.1001/archotol.131.6.481

[11] Pandya A, O’Brien A, Kovasala M, Bademci G, Tekin M, Arnos KS (2020) Analyses of del(GJB6-D13S1830) and del(GJB6-D13S1834) deletions in a large cohort with hearing loss: caveats to interpretation of molecular test results in multiplex families. Mol Genet genomic Med 8(4) 10.1002/mgg3.1171 PMC7196463 32067424

[12] del Castillo I, Villamar M, Moreno-Pelayo MA et al (2002) A deletion involving the connexin 30 gene in nonsyndromic hearing impairment. N Engl J Med 346(4):243–249 11807148 10.1056/NEJMoa012052

[13] Rodriguez-Paris J, Tamayo ML, Gelvez N, Schrijver I (2011) Allele-specific impairment of GJB2 expression by GJB6 deletion del(GJB6-D13S1854). PLoS One 6(6) 10.1371/journal.pone.0021665 PMC3126855 21738759

[14] Estivill X, Fortina P, Surrey S et al (1998) Connexin-26 mutations in sporadic and inherited sensorineural deafness. Lancet 351(9100):394–398 9482292 10.1016/S0140-6736(97)11124-2

[15] LeGrys VA, Yankaskas JR, Quittell LM, Marshall BC, Mogayzel PJ (2007) Diagnostic sweat testing: the Cystic Fibrosis Foundation guidelines. J Pediatr 151(1):85–89 17586196 10.1016/j.jpeds.2007.03.002

[16] Savant A, Lyman B, Bojanowski C, et al (1993) Cystic fibrosis. Pediatr Pulmonol. 1993;58(11):3013–3022. https://pubmed.ncbi.nlm.nih.gov/20301428/10.1002/ppul.26641 37594137

[17] Lilly E, Sellitto C, Milstone LM, White TW (2016) Connexin channels in congenital skin disorders. Semin Cell Dev Biol 50:4–12 26775130 10.1016/j.semcdb.2015.11.018 PMC4779425

[18] Richard G (2005) Connexin disorders of the skin. Clin Dermatol 23(1):23–32 15708286 10.1016/j.clindermatol.2004.09.010

[19] Sipos A, Vargas SL, Toma I, Hanner F, Willecke K, Peti-Peterdi J (2009) Connexin 30 deficiency impairs renal tubular ATP release and pressure natriuresis. J Am Soc Nephrol 20(8):1724–1732 19478095 10.1681/ASN.2008101099 PMC2723976

[20] Nakashima K, Kato H, Kurata R, et al (2023) Gap junction-mediated contraction of myoepithelial cells induces the peristaltic transport of sweat in human eccrine glands. Commun Biol 6(1) 10.1038/s42003-023-05557-9 PMC10657463 37980435

[21] Kutkowska-Kaźmierczak A, Niepokój K, Wertheim-Tysarowska K et al (2015) Phenotypic variability in gap junction syndromic skin disorders: experience from KID and Clouston syndromes’ clinical diagnostics. J Appl Genet 56(3):329–337 25575739 10.1007/s13353-014-0266-1 PMC4543413

[22] Meyer CG, Amedofu GK, Brandner JM, Pohland D, Timmann C, Horstmann RD (2002) Selection for deafness? Nat Med 8(12):1332–1333 12457154 10.1038/nm1202-1332

[23] Ilyaskin AV, Korbmacher C, Diakov A (2021) Inhibition of the epithelial sodium channel (ENaC) by connexin 30 involves stimulation of clathrin-mediated endocytosis. J Biol Chem 296 10.1016/j.jbc.2021.100404 PMC7973139 33577799

[24] Common JEA, Bitner-Glindzicz M, O’Toole EA et al (2005) Specific loss of connexin 26 expression in ductal sweat gland epithelium associated with the deletion mutation del(GJB6-D13S1830). Clin Exp Dermatol 30(6):688–693 16197390 10.1111/j.1365-2230.2005.01878.x

